# The New Georgia Dental Law

**Published:** 1885-11

**Authors:** 


ARTICLE VI.

### THE NEW GFORGIA DENTAL LAW.

[Approved and signed by the Governor, Oct. 9th. 1885.]

SECTION I. *Be it enacted by the General Assembly:*—That from and after the passage of this Act it shall be unlawful for any person to engage in the practice of dentistry in the State of Georgia, unless said person shall have obtained a license from a Board of Dentists, duly authorized and appointed under the provisions of this chapter, to issue license.

SEC. 2. That the Board of Examiners shall consist of five (5) dental graduates or practitioners of dentistry, who are members in good standing of the Georgia State Dental Society: provided, that said graduates or pratitioners have been practicing in the State of Georgia for a term of not less than three (3) years. Said Board shall be elected to serve for two years. The President of said Georgia State Dental Society shall have power to fill all vacancies in said Board for unexpired terms.

SEC. 3. It shall be the duty of this Board, first, to meet annually at the time of meeting of the Georgia State Dental Society, or oftener, at the call of any three of the members of said Board. Thirty days notice must be given of the annual meetings. Secondly, to prescribe a course of reading for those who study dentistry under private instructions. Thirdly, to grant license examination. Fourthly, to keep a book, in which shall be registered the names of all persons licensed to practice dentistry in the State of Georgia.

SEC. 4. That the book so kept shall be a book of record, and a transcript from it, certified to by the officer who has it in keeping, with the common seal, shall be evidence in any court in the state.

SEC. 5. That three members of said Board shall constitute a quorum for the transaction of business, and should a quorum not be present on the day appointed for their meeting, those present may adjourn from day to day until a quorum is present.

SEC. 6. That one member of said Board may grant a license to an applicant to practice until the next regular meeting of the Board, when he shall report the fact, at which time the temporary license shall expire, but such temporary licenses shall not be granted by a member of the Board after the Board has rejected the applicant.

SEC. 11. Repeals conflicting laws.

Approved October 9th, 1885.

---

### AN ACT.

To amend Section 1416 of the Code of Georgia relating to and regulating the Practice of Dentistry in the State of Georgia, and to require Practicing Dentists to register.

SECTION 1. *Be it enacted by the General Assembly:*—That section 1416 of the Code of Georgia be so amended as to read as follows: "That any person who shall, in violation of this Act, practice dentistry in the State of Georgia for a fee or reward shall be deemed guilty of a misdemeanor, and, upon conviction, shall be punished as prescribed in section 4310 of the Code of 1873; provided, that nothing in this Act shall be construed to prevent any person from extracting teeth; and provided further, that none of the provisions of this Act shall apply to regular licensed physicians and surgeons in practice at or prior to the passage of this Act, and dentists who were in practice prior to the 24th of August, 1872.

Sec. 2. Every person practicing dentistry in this State shall, within sixty days after the passage of this Act, register his name, together with his post-office and the date of his diploma or license, in the office of the Clerk of the Superior Court of the county in which he practices, and shall, on the payment to such Clerk of a fee of fifty cents, be entitled to receive from him a certificate of such registration.

Sec. 3. That all laws and parts of laws in conflict with this Act be, and the same are hereby, repealed.

Approved October 20, 1879.

